# The clinical and prognostic factors for biliary neuroendocrine neoplasm: a study based on the SEER database

**DOI:** 10.1186/s12893-022-01689-7

**Published:** 2022-06-29

**Authors:** Bo-Hao Zheng, Cheng Zhang, Wen-Ze Wan, Wen-Tao Sun, Xi Cheng, Xiao-Jian Ni, Xiao-Ling Ni, Tao Suo, Han Liu, Sheng Shen, Hou-Bao Liu

**Affiliations:** 1 Department of General Surgery, Zhongshan Hospital, Fudan University, 180 Fenglin Road, Shanghai, 200032 China; 2 Biliary Tract Disease Center, Zhongshan Hospital, Fudan University, 180 Fenglin Road, Shanghai, 200032 China; 3 Biliary Tract Disease Institute, Fudan University, 180 Fenglin Road, Shanghai, 200032 China; 4 Cancer Center, Zhongshan Hospital, 180 Fenglin Road, 200032 Shanghai, China; 5 Shanghai Engineering Research Center of Biliary Tract Minimal Invasive Surgery and Materials, Shanghai, China; 6 Department of General Surgery, Biliary Tract Disease Institute, Zhongshan Hospital, Fudan University, 180 Fenglin Road, Shanghai, 200032 China

**Keywords:** Neuroendocrine neoplasm, Biliary tract, Gallbladder, Prognosis, Propensity-matching analysis

## Abstract

**Background:**

In this study, we aimed at elucidating the postoperative survival and prognostic factors in patients with biliary neuroendocrine neoplasm (NEN).

**Methods:**

Cases of biliary system NEN and adenocarcinoma from 1975 to 2016 were extracted from the Surveillance, Epidemiology, and End Results (SEER) database. A propensity score matching (PSM) method was used to adjust baseline differences in clinicopathological characteristics in our analysis. The Kaplan–Meier analysis was carried out for survival analysis.

**Results:**

A total of 233 patients with biliary system NEN were enrolled in this study, of which 119 patients’ lesions located in gallbladder, while the others’ located in bile duct. The postoperative overall survival of bile duct NEN is significantly longer than that of gallbladder NEN (P < 0.001). For gallbladder NENs, surgery method (P = 0.020) and lymph node metastasis (P = 0.018) were identified as independent prognostic factors. In terms of ampulla of vater (AOV) NENs, age (P = 0.017) and lymph node metastasis (P = 0.006) were identified as independent prognostic factors, while grade (P = 0.002) and lymph node metastasis (P = 0.036) were identified as independent prognostic factors for extrahepatic bile duct (EBD) NENs. PSM analysis indicated that patients with biliary duct NENs have a better postoperative prognosis than biliary duct adenocarcinoma.

**Conclusions:**

Patients with NEN have better overall survival than patients with adenocarcinoma. Gallbladder NEN has an adverse prognosis than that of biliary tract NEN. The pathological subtype, differentiation, lymph node metastasis, surgery method, and lymph node resection could affect the postoperative prognosis of the gallbladder and biliary tract NEN.

**Supplementary Information:**

The online version contains supplementary material available at 10.1186/s12893-022-01689-7.

## Background

Neuroendocrine neoplasms (NENs), also known as carcinoid before, is a type of tumor that originate from peptidergic neural crest cells or neuroendocrine cells [1, 2]. NENs could express neuroendocrine markers and produce biologically active amines or hormonal peptides and are classified as “functional” or “non-functional” depending on whether the substances they produced cause-specific neuroendocrine-related symptoms [3, 4]. NENs can occur and develop in almost all organs of the human body. However, the characteristics, clinical manifestations, treatments, and prognosis of NENs vary with the locations and the pathological types [5, 6].

According to the WHO 2019 classification of tumors of the digestive system, Neuroendocrine Neoplasms (NENs) are classified into neuroendocrine tumors (NETs, which are well-differentiated), neuroendocrine carcinomas (NECs, which are poorly differentiated), and (mixed neuroendocrine–non-neuroendocrine neoplasms (MiNENs), which have neuroendocrine and non-neuroendocrine components simultaneously). In terms of the differentiation grade, the NETs are divided into G1/G2/G3, of which the mitotic rate (mitoses/2 mm^2^) is < 2/2–20/> 20, and the Ki-67 index is < 3%/3–20%/> 20%. Furthermore, the NECs could be divided into the small-cell type and the large-cell type [7, 8].

Biliary system NENs, especially those of the extrahepatic biliary tract, have a low incidence, which accounts for only 0.2–2% of all NENs [9]. According to the anatomy location, biliary NENs are further divided into gallbladder NENs and extrahepatic bile duct NENs (EBD-NENs), and the latter type includes the ampulla of vater NENs (AOV-NENs) [10]. Currently, the only curative therapeutic modality for biliary NENs is surgical resection, even though not all patients have well postoperative prognosis [11–13]

Limited to the rarity of biliary system NEN, the outcome and prognostic factors in patients have not been well elucidated. The application of large-scale database analysis may provide a basis for information and can help assess the relative efficacy of treatment options, further delineating the factors influencing overall survival (OS) [14, 15].

## Methods

### Cohort definition

Patients’ clinical information, clinicopathological features, and survival data are collected by searching data from 1975 to 2016 in the Surveillance, Epidemiology, and End Results (SEER) database using the National Cancer Institute SEER*Stat software (Version 8.3.6) by International Classification of Diseases for Oncology (ICD-O-3) code 8013/3, 8244/3, 8246/3, 8574/3 combined with site code C239-C241, C248-C249. SEER collects and publishes cancer incidence and survival data from 19 population-based cancer registries, covering approximately 34.6% of the U.S. population [16]. The selection criteria were as follows: (1) Patients without a history of previous anticancer therapy; (2) Patients without a history of other malignancies; (3) Patients receiving surgery after diagnosis; (4) Patients with pathologically proven biliary system neuroendocrine neoplasm.

Patients without complete survival time were excluded. Patients not receiving cancer-directed surgery were excluded, which means that in the SEER database, patients having the surgical codes of unknown or not performing cancer-directed surgery were excluded. Patients who were diagnosed only via death certificate or at autopsy were excluded. Other exclusion criteria included CS tumor size code of 999 (unknown or size not stated), 990 (microscopic focus or foci only and no size of focus is given), 000 (no mass/tumor found), and tumor grade unknown. A total of 233 patients were finally selected for the study.

### Parameter and group

According to the tumor’s anatomic location, there are mainly three groups: gallbladder, AOV, and bile duct. To investigate the clinicopathological characteristics of biliary NETs, the following information was obtained: age, gender, race, grade, histology, TNM stage, surgery treatment (surgery of primary site, the scope of regional LNs surgery), regional lymph nodes (LNs) status, tumor size and survival (months). All parameters were coded according to the SEER Program Coding and Staging Manual (2021 version). In this study, the tumor was staged according to the TNM stage system (AJCC 7th edition), and tumor grade is classified into grade I (Well differentiated), II (Moderately differentiated), III (Poorly differentiated), and IV (Undifferentiated), respectively.

### Propensity score matching analysis

To control for confounders, propensity score matching (PSM) was performed based on the logic of the propensity score and one-to-one nearest neighbor matching. The caliper was set as 0.02, and the ratio was 1:1. Six covariates (age, grade, Surgery method, lymph node metastasis, tumor size, and tumor number) were selected to calculate the propensity score. This analysis was carried out via the R package “Match it”.

### Statistical analysis

Overall survival (OS) was defined as the interval from the date of diagnosis until the date of death due to any cause or the last follow-up. Survival analysis was evaluated using the Kaplan–Meier method, and a log-rank test was used to assess any significant differences in OS stratified by each covariate.

The Cox proportional hazards models were used to analyze the associations between clinicopathological characteristics and patient survival. Hazard ratios (HR) and 95% confidence intervals (CI) were estimated using multivariate analysis.

The statistical analysis mentioned above and graphics were performed with SPSS software 20.0 (SPSS, Chicago, Illinois, USA) and GraphPad v7.0 (GraphPad). A two-tail P value ≤ 0.05 was considered statistically significant. R software (R Foundation for Statistical Computing, Vienna, Austria) was implicated in the PSM analysis.

## Results

### The baseline characteristics of patients

**Fig. 1:**
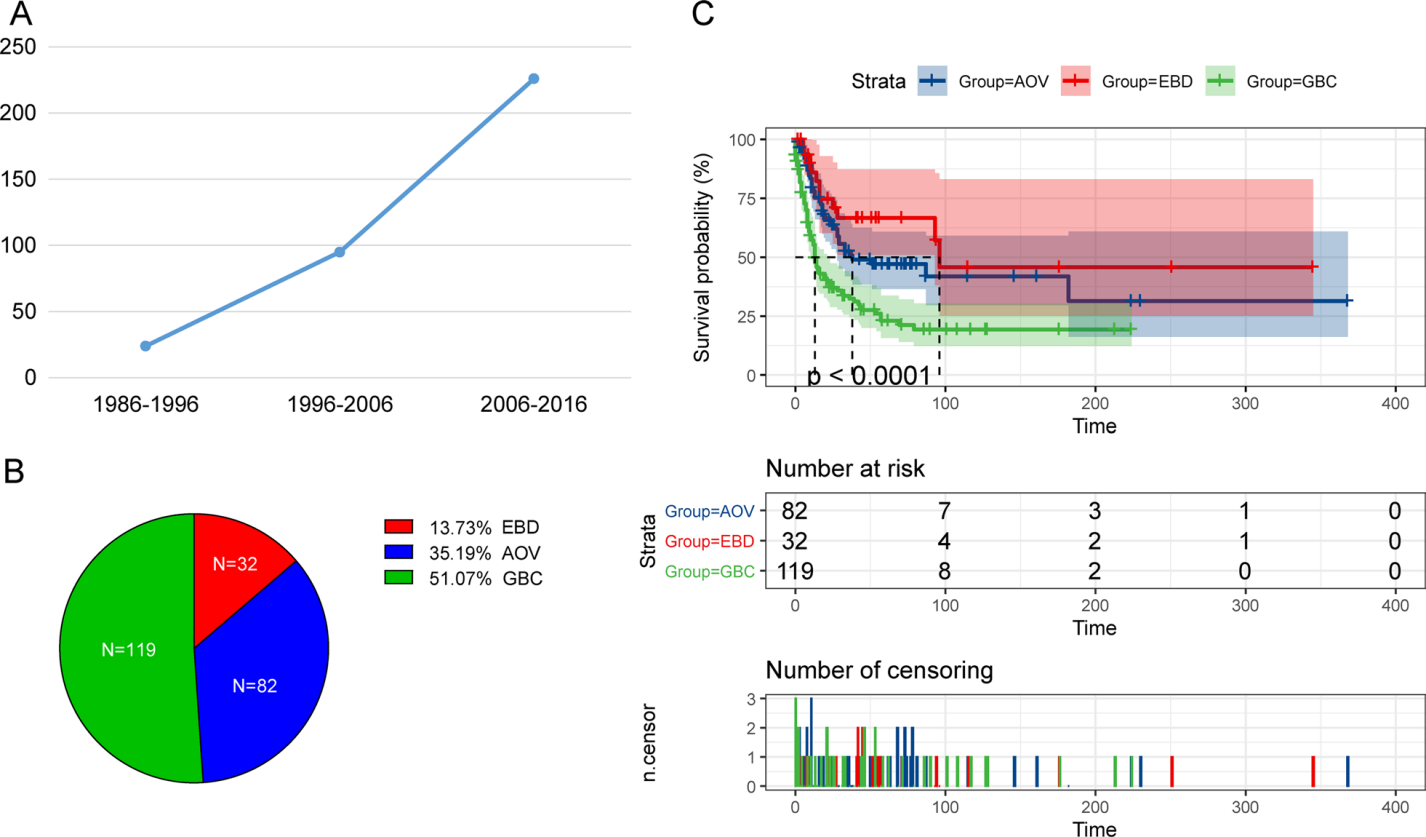
The overview of the bile system NENs from 1986 to 2016. **A** From 1986 to 2016, the incidence of bile system NEN is rising. **B** From 1986 to 2016, a total of 233 NEN patients received surgery. Among them, 119 patients (51.07%) patients were gallbladder NENs, 82 patients were AOV NENs, and 32 patients were EBD NENs. **C** The postoperative overall survival of biliary tract neuroendocrine neoplasm has better overall survival than that of the gallbladder neuroendocrine neoplasm (Log-rank test, P < 0.0001), while no significant differences were observed between the AOV NENs and EBD NENs in overall survival

Between 1975 and 2016, the incidence of the biliary system NENs is rising (Fig. 1A) and a total of 233 patients with biliary system NENs were diagnosed and received operations in the SEER database. Among them, 119 patients are diagnosed with gallbladder NENs, 82 patients are diagnosed as ampulla of vater (AOV) NENs, and 32 patients are diagnosed with extrahepatic bile duct (EBD) NENs (Fig. 1B). The detailed baseline characteristics are summarized in Table 1. Besides, survival analysis indicated that patients with biliary tract NEN, including AOV and EBD NENs, have better overall survival than that of patients with gallbladder NENs (P < 0.0001, Fig. 1C). This difference might be derived from the different tumor sites and treatment approaches.

**Table 1:** Baseline characteristics of patients with biliary system neuroendocrine neoplasm

Characteristics	Gallbladder (n = 119)	AOV (n = 82)	EBD (n = 32)
**Age at diagnosis, year**			
≤ 40	6	12	3
40–49	4	11	6
50–59	23	13	9
60–69	33	23	9
≥ 70	53	23	5
**Sex**			
Male	36	44	23
Female	83	38	9
**Race**			
White	97	67	21
Black	11	7	7
Others	11	8	4
**Grade**			
I	18	23	11
II	19	16	6
III	62	29	11
IV	20	14	4
**Pathology**			
NET	19	35	14
NEC	72	35	10
MiNEN	26	12	8
**RX Summ—Surg Prim Site (1998+)**			
Total surgical removal of primary site: enucleation (Total surgical removal of the primary site + Local tumor excision + Simple removal of primary site)	92	33	19
Radical surgery	27	49	13
**RX Summ—Scope Reg LN Sur (2003+)**			
None	79	13	12
1–3 regional lymph nodes removed	25	10	3
4 or more regional lymph nodes removed	15	59	17
**Regional nodes positive (1988+) (median, range)**	1 (0–15)	2 (0–14)	0 (0–6)
**Lymph node metastasis**			
Present	31	46	12
Absent	88	36	20
**CS tumor size (2004–2015) (median, range, mm)**	40 (3–150)	20 (2–90)	20 (2–65)
**Survival months (median, range, months)**	12 (0–224)	25.5 (0–368)	34.5 (2–345)

*AOV* ampulla of vater; *EBD* extrahepatic bile duct

### The survival analysis for biliary system NEN

**Fig. 2:**
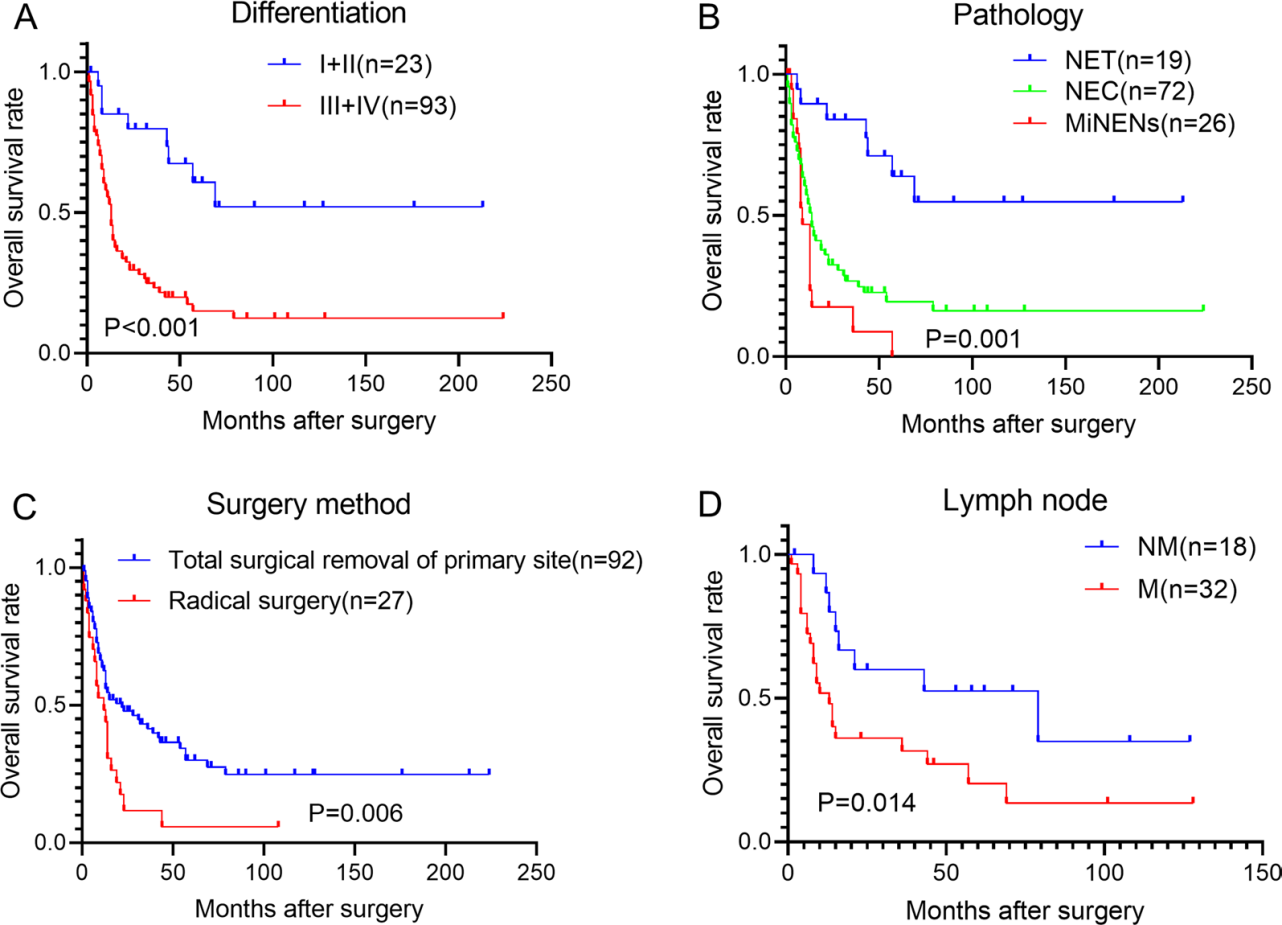
The postoperative prognosis factors for the gallbladder NENs. **A** Patients with higher differentiation levels (I + II) has better postoperative overall survival than those of the patients with lower differentiation levels (III+IV) (Log-rank test, P < 0.001). **B** The postoperative survival of NET was better than that of NEC and MiNENs (Log-rank test, P < 0.0001). **C** Patients receiving radical surgery have worse overall survival than patients receiving the local regional resection (Log-rank test, P = 0.006). **D** Patients with lymph node metastasis have worse postoperative overall survival than those of patients without lymph node metastasis (Log-rank test, P = 0.014)

To identify the factors influencing the postoperative survival of gallbladder NEN, Kaplan–Meier analysis was performed. We found poor differentiation level (P < 0.001, Fig. 2A), NEC (P < 0.0001, Fig. 2B), radical surgery approach (P = 0.006, Fig. 2C), and lymph node metastasis (P = 0.014, Fig. 2D), were associated with poor overall survival.

**Fig. 3:**
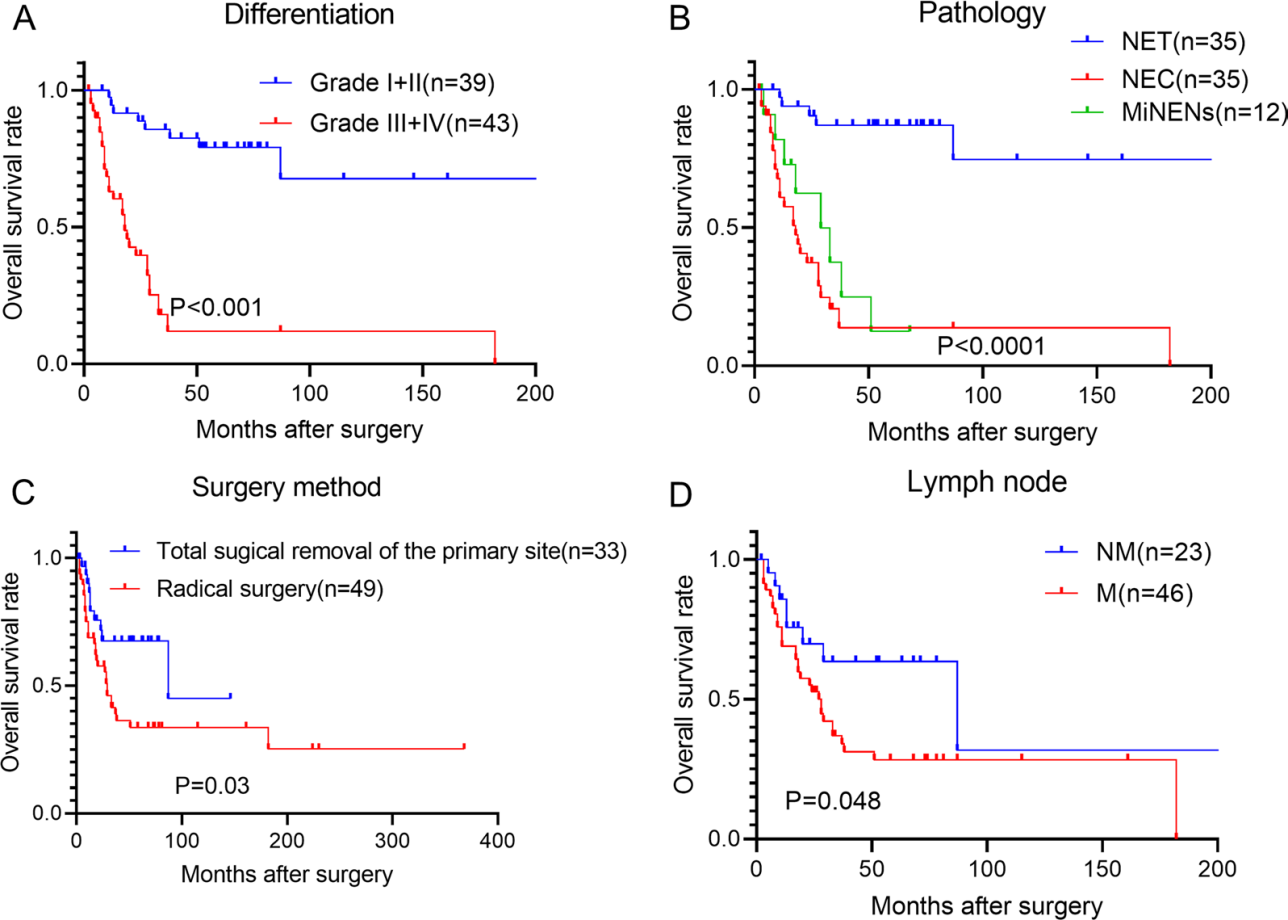
The postoperative prognosis factors for the AOV NENs. **A** The postoperative overall survival of patients with higher differentiation levels (I + II) has better overall survival than that of the patients with lower differentiation levels (III+IV) (Log-rank test, P < 0.001). **B** The postoperative survival of NET was better than that of NEC and MiNENs (Log-rank test, P < 0.0001). **C** Patients receiving radical surgery have worse overall survival than patients receiving the local regional resection (Log-rank test, P = 0.03). **D** Patients with lymph node metastasis have worse postoperative overall survival than those of patients without lymph node metastasis (Log-rank test, P = 0.048)

**Fig. 4:**
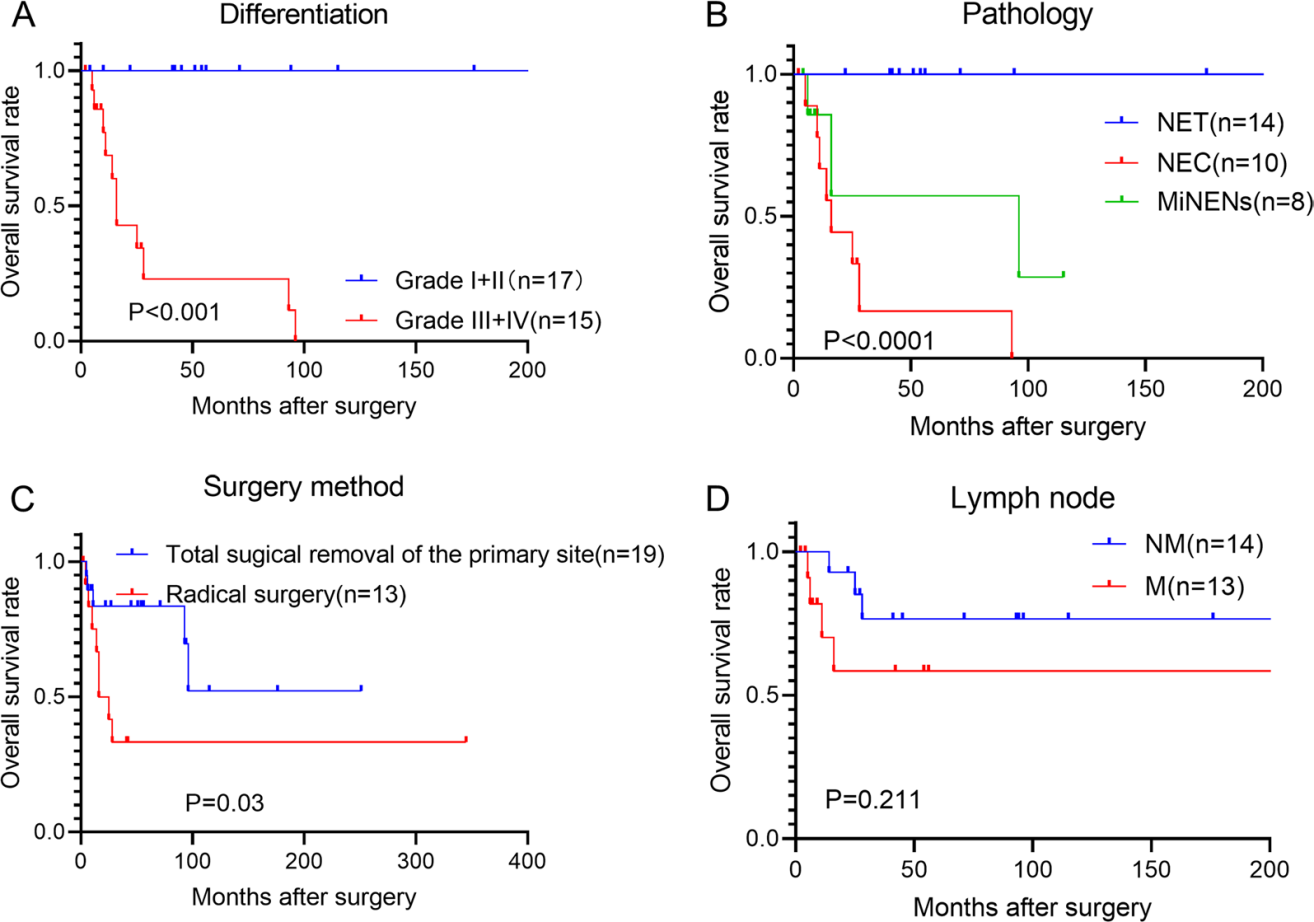
The postoperative prognostic factor for the EBD NENs. **A** The postoperative overall survival of patients with higher differentiation levels (I + II) was better than that of the patients with lower differentiation levels (III+IV) (Log-rank test, P < 0.001). **B** The postoperative survival of NET was better than that of NEC and MiNENs (Log-rank test, P < 0.0001). **C **Patients receiving radical surgery have worse overall survival than patients receiving the local regional resection (Log-rank test, P = 0.03). **D** Patients with lymph node metastasis have worse postoperative overall survival than those of patients without lymph node metastasis (Log-rank test, P = 0.211)

The same analyses were conducted in the biliary tract NEN. Similarly, we observed that poor differentiation level, lymph node metastasis, NEC, and radical surgery approach were associated with poor overall survival in both AOV (Fig. 3A–D) and EBD NENs (Fig. 4A–D).

Currently, the clinical value of lymph node resection is still under debate. In this study, we investigate the clinical value of lymph node resection in the treatment by evaluating the prognostic significance of the lymph node resection. Interestingly, lymph node resection didn’t achieve significant improvement in the postoperative overall survival in the gallbladder (P = 0.272), AOV (P = 0.203), and EBD NENs (P = 0.350) (Additional file 1). However, the statue of the lymph node metastasis is an important part of the tumor staging system and a significant prognostic factor. Herein, the lymph node resection should be performed to accurately stage the NEN patients.

### The multivariate prognostic factors for gallbladder NENs and biliary NENs

To identify the independent postoperative factors for NENs, multivariate analyses were performed. In gallbladder NENs, surgery method, and lymph node metastasis were identified as independent prognostic factors (Table 2). Meanwhile, in AOV NENs, age, and lymph node metastasis were identified as independent prognostic factors (Table 2). Besides, in EBD NENs, tumor grade and lymph node metastasis were identified as independent prognostic factors (Table 2). All these results indicated that lymph node metastasis status was an important prognostic factor for biliary system NEN patients.

**Table 2:** The univariate and multivariate analysis for biliary system NEN

Variable	Univariate analysis	Multivariate analysis		
	*P* value	Hazard ratio (HR)	95% confidence interval (CI)	*P* value
Gallbladder NEN				
Age	0.081	/	/	/
Race	0.516	/	/	/
Gender	0.699	/	/	/
Grade	**0.001**			0.641
Pathology	**0.017**			0.662
Surgery method	**0.023**	3.397	1.209–9.541	**0.020**
Lymph node metastasis	**0.027**	4.236	1.276–14.059	**0.018**
Tumor size	**0.036**			0.490
AOV NEN				
Age	0.010	2.198	1.150–4.199	**0.017**
Race	0.146			
Gender	0.699			
Grade	**P < 0.001**			0.239
Pathology	**0.024**			0.220
Surgery method	**0.048**			0.944
Lymph node metastasis	**0.004**	2.633	1.312–5.285	**0.006**
Tumor size	0.773			
EBD NEN				
Age	0.537	/	/	/
Race	0.274	/	/	/
Gender	0.330	/	/	/
Grade	**0.001**	7.821	2.122–28.828	**0.002**
Pathology	0.062	/	/	/
Surgery method	0.380	/	/	/
Lymph node metastasis	**0.036**	6.141	1.169–32.244	**0.032**
Tumor size	**0.015**	/		/

*P* values less than 0.05 are shown in bold

### PSM analysis

**Fig. 5:**
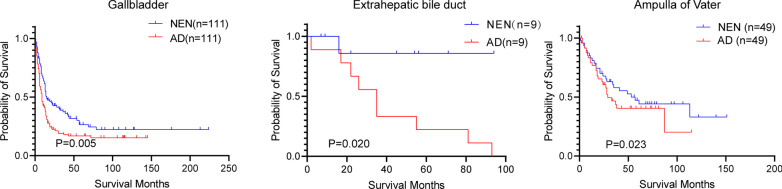
PSM analysis indicated that biliary system neuroendocrine neoplasm (NEN) has better survival than that of biliary system adenocarcinoma (AD)

To evaluate the postoperative overall survival rate between the biliary system adenocarcinoma and biliary system NEN, 4216 gallbladders, 394 extrahepatic bile duct, and 3046 ampulla of vater adenocarcinoma were selected for the comparison. To reduce the clinical differences between the different groups, PSM analysis was carried out, then Kaplan–Meier analysis was carried out to evaluate the overall survival differences. In the gallbladder, 111 pairs of patients were selected through the PSM analysis, and the Kaplan–Meier analysis indicated that gallbladder NEN patients have a better overall rate than that of gallbladder adenocarcinoma (P = 0.005, Fig. 5). Similar results were observed for the EBD (P = 0.020) and AOV (P = 0.023) neoplasms, after the adjustment of the PSM (Fig. 5). All these data indicated that regardless of the location of the tumor, biliary system NENs have better postoperative overall survival than that of biliary system adenocarcinoma.

## Discussion

With the improvement in the diagnostic tools, the incidence and prevalence of NENs are increasing in recent years. In general, the majority of the NENs are located in the lung and pancreas, while biliary system NENs are very rare forms of NENs. Therefore, the clinicopathologic features and prognosis of patients with biliary NENs remain unclear.

In this study, a total of 233 biliary system NEN patients were retrieved from the SEER database. Our analyses indicated that gallbladder NENs have worse overall survival than that biliary NENs. Besides, our study identified independent prognostic factors for gallbladder, AOV, and EBD NEN patients, respectively. Finally, our PSM data indicated that NENs in biliary systems have better overall survival than that of adenocarcinoma in biliary systems.

Consistent with previous studies [17, 18], after the PSM, our data showed that the prognosis of NENs is better than that of adenocarcinoma, which might be derived from the origination of NENs is different from the origination of the adenocarcinoma. This difference could result in different biological characteristics, thus inducing different clinical outcomes. Besides, recent studies indicated that poorly differential NENs had a good response to the chemotherapy, which might explain the better OS of patients with NENs [19, 20]. Moreover, recent studies have reported that gastric NEC has unique clinicopathological features quite different from intestinal-type gastric cancer (IGC) and may have a superior survival to IGC in early-stage patients, indicating that NENs have better survival than other pathological types of cancers, like adenocarcinoma [21, 22], which is consistent with our research.

In the survival analyses, considering the tumor location, the prognosis of the gallbladder NEN was significantly worse than that of the AOV and EBD NEN. It is might because the anatomical location and anatomical characteristics of the gallbladder made the gallbladder NEN more prone to invasion and infiltration of the liver and its surrounding structures, and gallbladder NEN was often diagnosed at a late stage in its course due to occult symptoms [23], while the AOV or EBD NEN was more likely to induce clinical symptoms like jaundice, making it possibly discovered and diagnosed at the early stage [24]. Therefore, cultivating good health awareness, regular health checkups, and the application of more precise diagnostic techniques could increase the early diagnosis rate of gallbladder NEN, thereby improving the prognosis of patients with gallbladder NEN [25]. Besides, the biological characteristics of NEN could vary with the location of the tumor, which might cause differences among the prognosis of the gallbladder, AOV, and EBD NENs. For instance, an analysis based on the SEER database indicates that colon NECs (co-NECs) frequently originate on the right side and commonly develop liver metastasis and right-sided co-NECs are associated with better survival than left-sided co-NECs after liver metastasis has occurred [26]. In addition, a recent study indicated that the prognosis of gastroenteropancreatic NETs (GEP-NETs) varies with the tumor site. For instance: patients with rectal or appendiceal GEP-NETs had the best median OS among site groups, whereas patients with GEP-NETs in the pancreas had the worst median OS [27].

For patients with bile system NEN, there was no significant difference in overall survival between cases with and without lymphadenectomy. However, patients with lymph node tumor infiltration obtained a worse prognosis. Therefore, for patients with biliary NEN, lymph node metastasis should be fully assessed when considering whether lymph node resection should be performed.

The present study suggested that age at diagnosis, grade, AJCC TNM stage, AJCC stage group, tumor size, histology, lymph node metastasis, etc. were independent prognostic factors of OS in biliary tract cancer [28–30]. Limited by the data content, we analyzed prognostic factors based on some of these factors. Our study indicated that the independent prognostic factor varies with the location tumor. For GB-NENs, surgery method and lymph node metastasis were identified as independent prognostic factors. In terms of AOV NENs, age and lymph node metastasis were identified as an independent prognostic factors, while grade and lymph node metastasis were identified as independent prognostic factors for EBD NENs.

There are several limitations in this study. In this study, we analyzed the prognosis of NENs located at AOV, EBD, and gallbladder separately according to the tumor site, resulting in a small number of samples per data set. A large cohort is needed to validate our results. Moreover, merely using the database as a data source will cause the loss of the validation set, which meant the necessity of some real-world research to validate our results. Unfortunately, many clinicopathological features such as TNM stage and ki67 are missing in the SEER database. Herein, the analysis of the prognostic factors is incomplete. Meanwhile, in this study, patients’ statuses of multimodality therapies such as radiotherapy, and chemotherapy were not taken into consideration. The acquisition of higher-quality data and the development of larger sample sizes might help us get better prognostic prediction models.

## Conclusions

In brief, although many independent factors can affect the prognosis of NENs of the biliary system, NENs of the biliary system are a disease with a poor prognosis. New treatment methods and more reasonable management methods are urgently needed to be explored.

## Supplementary Information

**Additional file 1: ** The overall survival of patients who did/didn’t undergo lymph node resection. **A**–**C** The postoperative overall survival of patients who underwent lymph node resection was not better than those of patients who didn’t undergo lymph node resection (P = 0.272 in gallbladder NENs, P = 0.203 in AOV NENs, P = 0.350 in EBD NENs).

## Data Availability

The datasets generated and analysed during the current study are available in the Surveillance, Epidemiology, and End Results (SEER) database repository, https://seer.cancer.gov/.

## Abbreviations

NEN: Neuroendocrine neoplasm
SEER: Surveillance, Epidemiology, and End Results
PSM: Propensity score matching
AOV: Ampulla of vater
EBD: Extrahepatic bile duct
NET: Neuroendocrine tumors
NEC: Neuroendocrine carcinomas
MiNENs: Mixed neuroendocrine–non-neuroendocrine neoplasms
LNs: Lymph nodes
OS: Overall survival
CI: Confidence intervals
HR: Hazard ratios
