# Supplementary figures and images for: The clinical and prognostic factors for biliary neuroendocrine neoplasm: a study based on the SEER database
Source: BMC Surg. 2022 Jun 29;22:253. doi: 10.1186/s12893-022-01689-7 (PMC9245279; doi:10.1186/s12893-022-01689-7)

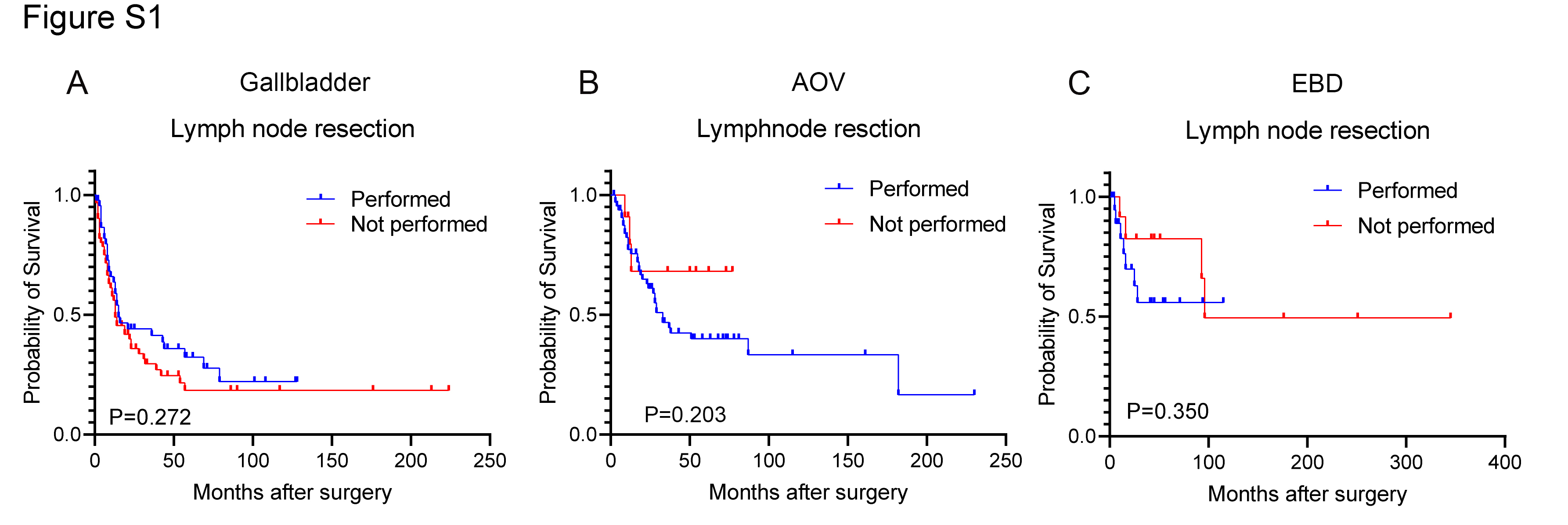

Supplement: Supplementary file 1 — Additional file 1: The overall survival of patients who did/didn’t undergo lymph node resection. A–C The postoperative overall survival of patients who underwent lymph node resection was not better than those of patients who didn’t undergo lymph node resection (P = 0.272 in gallbladder NENs, P = 0.203 in AOV NENs, P = 0.350 in EBD NENs). [file 12893_2022_1689_MOESM1_ESM.jpg]
